# Supplementary material for: Shotgun Proteomics of Isolated Urinary Extracellular Vesicles for Investigating Respiratory Impedance in Healthy Preschoolers
Source: Molecules. 2021 Feb 26;26(5):1258. doi: 10.3390/molecules26051258 (PMC7956503; doi:10.3390/molecules26051258)
Supplement: Supplementary file 1 [file molecules-26-01258-s001.zip › Revised_molecules_1045645/Supplementary Figures captions.docx]

**Supplementary Figures captions**

**Figure S1.** Mapping and repeatability of urinary extracellular vesicles by proteomics analysis. a) Virtual 2Dmap of identified proteins, according to their pI and MW (Dalton); a color/shape code was assigned for each protein according to the confidence of SEQUEST score values (yellow/triangle ≤ 15, blue/square 15-35 and red/circle ≥ 35). b) Typical technical repeatability (LP7 sample).

**Figure S2.** Proteins shared among subgroups obtained by clustering. Proportional Venn diagram of protein distribution in the three stratified groups: A, B and C.

**Figure S3**. Enrichment analysis of proteins identified in the three groups by nLC-MS/MS. a)Venn diagrams illustrating the comparison of proteins detected in the three analysed groups versus Vesiclepedia database (group A = green, group B = blue, group C = red). For the overlay analysis, all gene entries from Vesiclepedia were downloaded, imported in Funrich and filtered for urine studies (5064 entries on 72 experiments selected from the entire database). b) Enrichment analysis of cellular components of all identified proteins in the three analysed groups. Using FunRich tool, enriched gene ontology terms are shown in bar charts using a double y-axis plot. Y-axis in left indicates the percentage of genes enriched for each reported cellular component (represented using green bars for group A, blue bars for group B and red bars for group C) and y-axis in right indicates the corresponding –log10 p values BH corrected (marked using light green line for group A, light blue line for group B and orange line for group C). Only gene ontology terms with a p value < 1E-4 are reported.

**Figure S4.** Volcano-like plot using Dave and DCI values of proteins with p-value < 0.01 from LDA; see Supplementary table 3. To simplify graph, DCI value is reported as Log10[|DCI|]. Dashed lines correspond to the fixed DAVE and DCI limits (|0.2| and |5|, respectively). It is possible to observe that in group A more proteins are up-regulated compared to group C; similar trend was into the comparison B vs C groups.

**Figure S5.** Calculation of α-value for each subject. Typing of group through MS-based α-value algorithm in the 21 patients analyzed.

**Figure S6.** Identification of protein pathways, in the three groups A, B and C.
