# Supplementary figures and images for: Shotgun Proteomics of Isolated Urinary Extracellular Vesicles for Investigating Respiratory Impedance in Healthy Preschoolers
Source: Molecules. 2021 Feb 26;26(5):1258. doi: 10.3390/molecules26051258 (PMC7956503; doi:10.3390/molecules26051258)

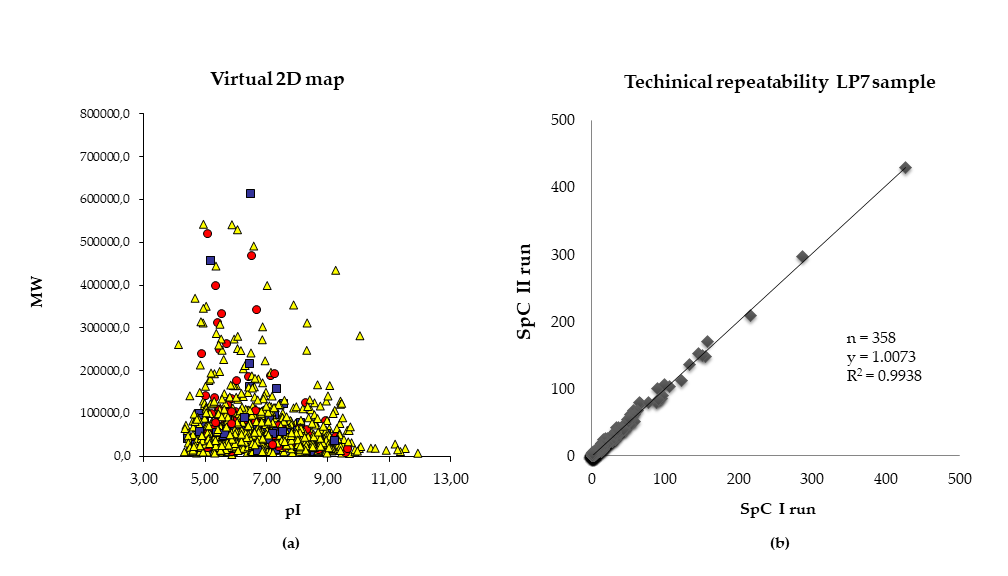

Supplement: Supplementary file 1 [file molecules-26-01258-s001.zip › Revised_molecules_1045645/Figure S1.tif]

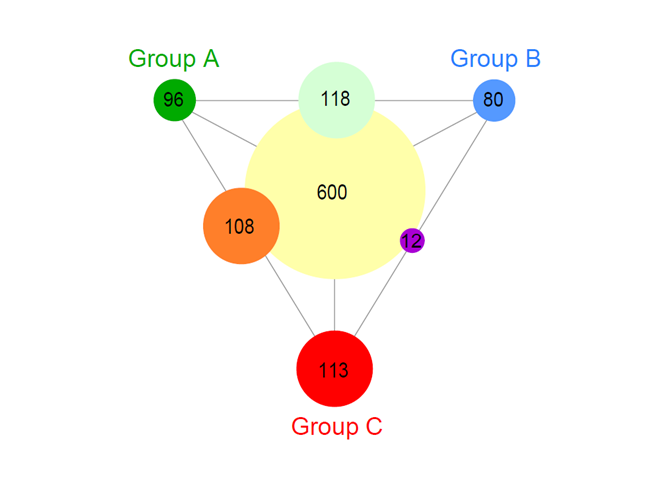

Supplement: Supplementary file 1 [file molecules-26-01258-s001.zip › Revised_molecules_1045645/Figure S2.tif]

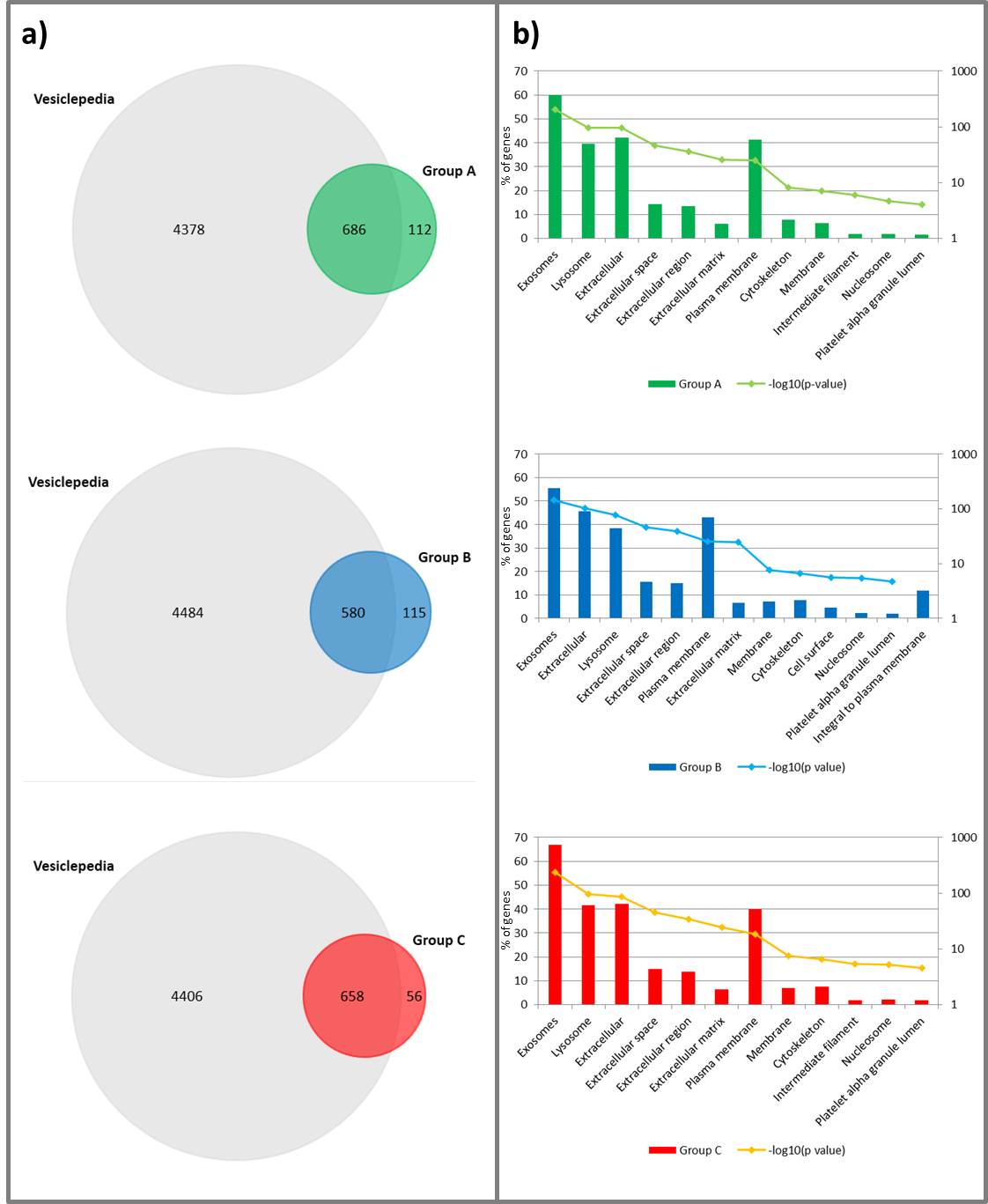

Supplement: Supplementary file 1 [file molecules-26-01258-s001.zip › Revised_molecules_1045645/Figure S3_New.jpg]

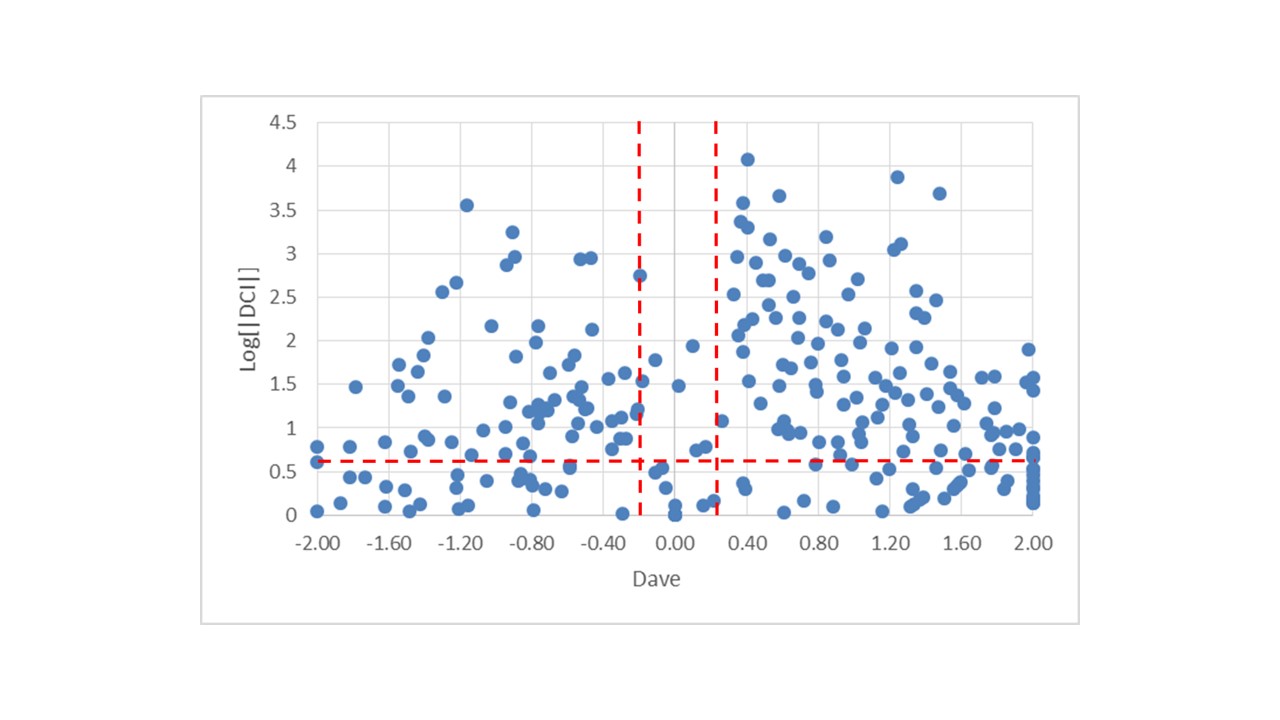

Supplement: Supplementary file 1 [file molecules-26-01258-s001.zip › Revised_molecules_1045645/Figure S4_New.jpg]

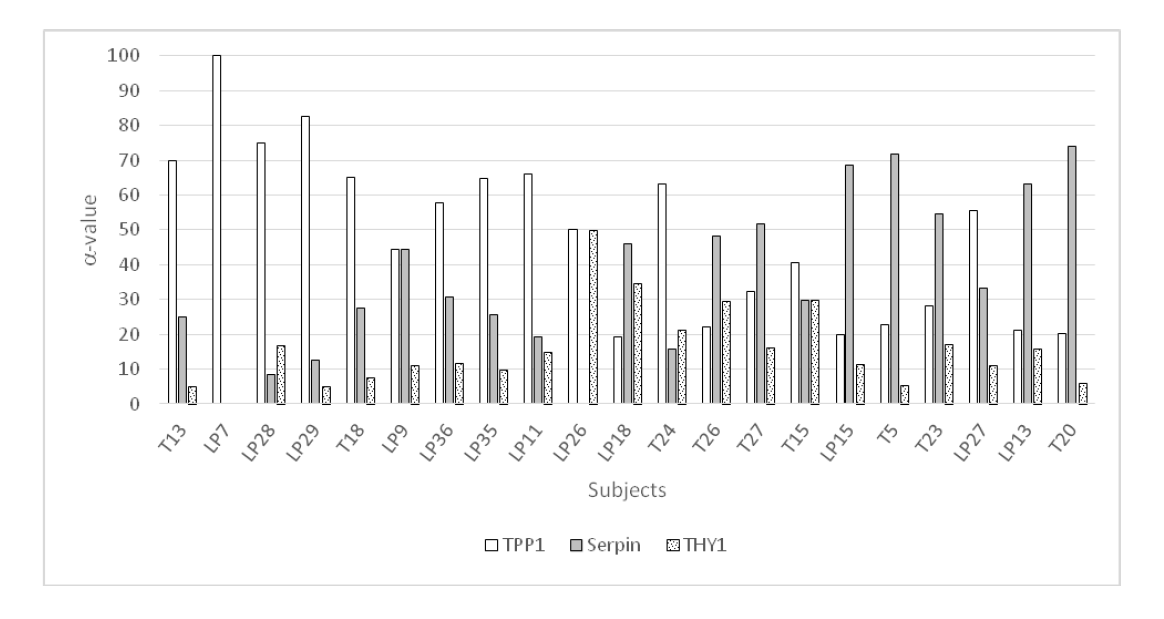

Supplement: Supplementary file 1 [file molecules-26-01258-s001.zip › Revised_molecules_1045645/Figure S5_Ex Figure 3.tif]
