# Supplementary material for: Shotgun Proteomics of Isolated Urinary Extracellular Vesicles for Investigating Respiratory Impedance in Healthy Preschoolers
Source: Molecules. 2021 Feb 26;26(5):1258. doi: 10.3390/molecules26051258 (PMC7956503; doi:10.3390/molecules26051258)
Supplement: Supplementary file 1 [file molecules-26-01258-s001.zip › Revised_molecules_1045645/Supplementary Materials.doc]

**Supplementary Materials**

**Participants and study design**

The current study is part of the ongoing longitudinal observational study “PREmaturely born preschool children-Asthma and Allergic Rhinitis” (PRE-AR) conducted at the Pediatric Pulmonology Outpatient Clinic of the Institute for Research and Biomedical Innovation (IRIB) of the National Research Council (CNR) in Palermo.

**Extracellular Vesicle sample preparation**

The urine samples (100 ml) were collected in the morning from 21 children (12 preterm born children and 9 term born children). The urine was centrifuged at 17,000 x g for 10 min at 4°C in order to remove sediment including whole cells, large membrane fragments, and other debris. Supernatants were splitted in 5 ml fractions and subjected to ultracentrifugation at 200,000 x g for 1h at 4°C to obtain extracellular vesicle pellets. The pellets were resuspended in 0.1 M ammonium bicarbonate, pH 7.9, and treated with RapiGest SF reagent (Waters Corporation, Milford, MA, USA) at the final concentration of 0.25% (w/v). After incubation at 100°C for 20 min, the samples were cooled to room temperature and centrifuged at 2,200 x g for 10 min. Subsequently, the protein concentration was assayed using the SPN^TM^ Protein Assay kit, and 50±0.5 µg of proteins from each sample were digested with trypsin (Promega, Madison, WI, USA), using a 1:50 (w/w) enzyme/substrate ratio, at 37°C overnight. The next morning, an additional aliquot of trypsin was added at an enzyme/substrate ratio of 1:100 (w/w) and digestion continued for 4 h. Enzymatic digestion was stopped with the addition of 0.5% Trifluoroacetic Acid (TFA) (Sigma-Aldrich Inc., St.Louis, MO, USA), and a subsequent incubation at 37°C for 45 minutes completed the RapiGest acid hydrolysis. Water immiscible degradation products were removed by centrifugation at 13,000 rpm for 10 minutes. Finally, samples were desalted by Pierce^TM^ C-18 spin columns (Thermo Fisher Scientific, Waltham, MA, USA), concentrated in a SpeedVac (Savant Instruments Farmingdale, NY, USA) at 60°C and resuspended in 0.1% formic acid (Sigma-Aldrich Inc., St.Louis, MO, USA) at a concentration of 1µg/µL.

**Proteomic analysis**

Trypsin-digested mixtures were analyzed using the Eksigent nanoLC-Ultra 2D System (Eksigent, AB SCIEX Dublin, CA, USA) combined with the cHiPLC-nanoflex system (Eksigent) in trap-elute mode. Briefly, samples were first loaded on the cHiPLC trap (200 μm x 500 μm ChromXP C18-CL, 3 μm, 120 Å) and washed in isocratic mode with 0.1% aqueous formic acid for 10 min at a flow rate of 3 μL/min. The automatic switching of a cHiPLC ten-port valve then eluted the trapped mixture on a nano cHiPLC column (75 μm x 15 cm ChromXP C18-CL, 3 μm, 120 Å), through a 65 min gradient of 5–45% of eluent B (eluent A, 0.1% formic acid in water; eluent B, 0.1% formic acid in acetonitrile), at a flow rate of 300 nL/min. To preserve system stability, in terms of elution times of components, trap and column were maintained at 35°C.

Mass spectra were acquired using a QExactive mass spectrometer (Thermo Fisher Scientific, San José, CA, USA), equipped with an EASY-Spray ion source (Thermo Fisher Scientific, San Josè, CA, USA). Easy spray was achieved using an EASY-Spray Emitter (Dionex Benelux BV, Amsterdam, The Netherlands) (nanoflow 7 µm ID Transfer Line 20µm x 50 cm) held to 2.1 kV, while the ion transfer capillary was held at 220 °C. Full mass spectra were recorded in positive ion mode over a 400-1600 m/z range with a resolution setting of 70000 FWHM (@ m/z 200) with 1 microscan per sec. Each full scan was followed by 10 MS/MS events, acquired at a resolution of 17,500 FWHM, sequentially generated in a data-dependent manner on the top ten most abundant isotope patterns with charge ≥2, selected with an isolation window of 2 m/z for the survey scan, fragmented by higher energy collisional dissociation (HCD) with normalized collision energies of 30 and dynamically excluded for 5 s. The maximum ion injection times for the survey scan and the MS/MS scans were 50 and 200 ms and the ion target values were set at 106 and 105, respectively.

**Proteomics Data Processing**

All data generated were searched using the Sequest search engine contained in the Thermo Scientific Proteome Discoverer software, version 2.1. The experimental MS/MS spectra were correlated to tryptic peptide sequences by comparison with the theoretical mass spectra obtained by *in silico* digestion of the human protein database (about 70726 entries), downloaded January 2017 from the UNIPROT website ([www.uniprot.gov](http://www.ncbi.nlm.nih.gov/)). The following criteria were used for identification of peptide sequences and related proteins: trypsin was used as an enzyme; three missed cleavages per peptide were allowed; and mass tolerances of 10 ppm for precursor ions and ±0.05 Da for fragment ions were applied. A percolator node was used with a target-decoy strategy to give final false discovery rates (FDR) at Peptide Spectrum Match (PSM) level of 0.01 (strict) based on q-values, considering maximum deltaCN of 0.05^1^. Only peptides with high confidence, minimum peptide length of six amino acids, and rank 1 were considered. Protein grouping and strict parsimony principle were applied.

**Proteomics data-mining and label-free quantification**

The protein lists obtained as output data from the Sequest search were compared with a label-free quantification approach. Specifically, the protein lists considered were aligned and then processed by means of Linear Discriminant Analysis (LDA)^2^. A matrix mxn consisting of 1127 proteins and 21 samples grouped into three different categories of children (Group A, n=9; Group B, n=6; Group C, n=6) was considered. LDA was applied by using a common covariance matrix for all groups, and the Mahalanobis distance^3^ was measured from each point to each group’s multivariate mean. To select proteins discriminating the analyzed groups of children (A, B and C), we considered those with the largest F ratio (≥5) and smallest *p*-value (≤0.01). Based on a direct correlation between the SpC and the relative abundance of the identified proteins, DAve (Differential Average) and DCI (Differential Coefficient Index) indices of the MAProMa (Multidimensional Algorithm Protein Map) software^4^ were used to process the average spectral counts (aSpC) corresponding to each analyzed group of children (A, B and C). Specifically, the threshold values imposed were DAve >|0.2| and DCI>| 5|. In addition, the average spectral count (aSpC) was evaluated by t-test, too^5^. The average Spectral Count (aSpC) values of proteins selected by LDA were pairwise compared by DAve and DCI indices of MAProMa. Finally, proteins selected by both LDA and MAProMA were evaluated by hierarchical clustering^6^ applying Ward*’s* method and the Euclidean distance metric.

**Definition of α-value**

Spectral count (i.e. the number of MS/MS spectra attributed to a protein) was assumed as a semi-quantitative parameter for calculating the α-values of the identified group-specific proteins (*i* =A or B κ or C)^7,8^. The α-value is a normalized parameter obtained by the following formula:

αi,j=100*(Pi,j/Σ(i=1→3)Pi,j)

where:

Pi,j = spectral count of a biomarker i in a subject j

i = selected biomarker proteins (TPP1, SERPIN, and THY1 for groups A, B and C, respectively).

Because the sum of the 3 α-values within each subject is equal to 100, an α-value of > 50 may be used to identify the specific group related to the predominant biomarker.

**Label-free quantitation**

*Linear discriminant analysis*

A matrix *m*x*n* consisting of 1127 proteins and 21 samples grouped into three different groups of children (A, B, C) was considered. LDA was applied using a common covariance matrix for all groups, and the Mahalanobis distance^3^ from each point to each group’s multivariate mean; to select proteins discriminating the groups of children analyzed we considered those with the largest F ratio (≥5) and smallest p-value (≤0.01).

*DAve and DCI evaluation*

Based on a direct correlation between SpC and the relative abundance of the identified proteins, DAve and DCI indices were used to process the average spectral count (aSpC) corresponding to each analysed group of children (A, B,C). The analysed groups were pairwise compared (A vs. C, B vs. C, A vs. B).

In detail, DAve, which evaluates changes in protein expression, was defined as:

(X-Y)/(X+Y)/0.5

while DCI, which evaluates the confidence of differential expression, was defined as:

(X+Y)*(X-Y)/2

where X and Y represent the aSpC of a given protein in two compared samples. To identify proteins whose expression significantly changed in a given pairwise comparison, proteins with DAve ≥|0.2| and DCI>|5| were retained. In addition, the DAve value of proteins identified exclusively in one of the two compared samples was set to +2.

**References**

1. Käll, L.; Canterbury, J.D.; Weston, .J; Noble, W.S.; MacCoss, M.J. Semi-supervised learning for peptide identification from shotgun proteomics datasets. *Nat Methods.* **2007**, *4*, 923-925.
2. Hilario, M.; Kalousis, A. Approaches to dimensionality reduction in proteomic biomarker studies. *Brief Bioinform.* **2008**, *9*, 102–118.
3. Jain, A.K.;Murty, M.N., Flynn, P.J. Data Clustering: A Review. *ACM Comput Surv.* **1999**, *31*, 264–323.
4. Mauri, P.; Dehò, G. Proteomic Approach to the Analysis of RNA Degradosome Composition in Escherichia coli. *Methods Enzymol.* **2008**, *447*, 99-117. doi: 10.1016/S0076-6879(08)02206-4.
5. Zhang, B., VerBerkmoes, N.C., Langston, M.A.; Uberbacher, E.; Hettich, R.L.; Samatova, N.F. Detecting differential and correlated protein expression in label-free shotgun proteomics. *J Proteome Res.* ***2006***, *5*, 2909–2918
6. Zhao, Y.; Karypis, G. Data clustering in life sciences. *Mol Biotechnol* **2005,** *31*, 55–80.
7. Mauri, P.L.; Scigelova M. Multidimensional protein identification technology for clinical proteomic analysis. *Clin Chem Lab Med.* **2009,** 47, 636-646.
8. Brambilla, F.; Lavatelli, F.; Di Silvestre, D.; Valentini, V.; Rossi, R.; Palladini, G.; Obici, L.; Verga, L.; Mauri, P.; Merlini, G. Reliable typing of systemic amyloidoses through proteomic analysis of subcutaneous adipose tissue. *Blood.* **2012**, *119*, 1844-1847.
