# Supplementary material for: Shotgun Proteomics of Isolated Urinary Extracellular Vesicles for Investigating Respiratory Impedance in Healthy Preschoolers
Source: Molecules. 2021 Feb 26;26(5):1258. doi: 10.3390/molecules26051258 (PMC7956503; doi:10.3390/molecules26051258)
Supplement: Supplementary file 1 [file molecules-26-01258-s001.zip › Revised_molecules_1045645/Table S1.doc]

|  | All  n=66 | Sub sample  n=21 | Rest of the sample  n=45 |  | p-value* |
| --- | --- | --- | --- | --- | --- |
| *Personal characteristics* |  |  |  |  |  |
| Female | 31 (46.96%) | 7 (33.33%) | 24 (53.33%) |  | 0.21 |
| Z-score Height, mean (SD) | -0.51 (1.06) | -0.44 (1.18) | -0.55 (1.01) |  | 0.69 |
| Z-score BMI, mean (SD) | 0.97 (1.18) | 0.83 (1.31) | 1.04 (1.12) |  | 0.50 |
| Age, *years*, mean (SD) | 4.1 (0.84) | 4.05 (0.86) | 4.12 (0.83) |  | 0.75 |
| Birthweight, mean (SD), gr | 2488.09 (756.91) | 2504.29 (813.05) | 2480.53 (738.71) |  | 0.76 |
| Cesarean Delivery, no. (%) | 53 (80.30%) | 17 (80.95%) | 36 (80.00%) |  | 1.00 |
| Maternal diseases in pregnancy, no. (%) | 8 (12.21%) | 2 (9.52%) | 6 (13.33%) |  | 0.97 |
| Received oxygen at any time, n (%) | 26 (39.39%) | 8 (38.10%) | 18 (40.00%) |  | 0.97 |
| Bronchiolitis 1st year of life, n (%) | 22 (33.85%) | 8 (40.0%) | 14 (31.11%) |  | 0.68 |
| Upper respiratory infection ever, n (%) | 30 (45.45%) | 9 (42.86%) | 21 (46.67%) |  | 0.98 |
| Pneumonia ever, n (%) | 15 (22.73%) | 3 (14.29%) | 12 (26.67%) |  | 0.42 |
| *Environmental exposure* |  |  |  |  |  |
| Proximity to high traffic road < 200m, n (%) | 55 (87.87%) | 19 (90.47%) | 39 (86.77) |  | 0.97 |
| Current parental smoke exposure, n (%) | 11 (16.67%) | 4 (19.05%) | 7 (15.56%) |  | 1.00 |
| Current pet exposure, n (%) | 10 (15.15%) | 5 (23.81%) | 5 (11.11%) |  | 0.33 |
| Current mold exposure, n (%) | 11 (16.67%) | 2 (9.52%) | 9 (20%) |  | 0.48 |
| *FOT parameters* |  |  |  |  |  |
| Z-score |  |  |  |  |  |
| Rrs6 | 0.59 (1.83) | 0.24 (2.76) | 0.76 (1.15) |  | 0.625 |
| Rrs8 | 0.38 (1.87) | -0.02 (2.83) | 0.57 (1.16) |  | 0.797 |
| Rrs10 | 0.51 (1.88) | 0.12 (2.83) | 0.69 (1.18) |  | 0.862 |
| Xrs6 | -0.72 (1.94) | -0.69 (2.15) | -0.74 (1.86) |  | 0.602 |
| Xrs8 | -0.82 (1.68) | -1.13 (2.28) | -0.67 (1.31) |  | 0.182 |
| Xrs10 | -0.74 (1.66) | -1.01 (2.10) | -0.61 (1.40) |  | 0.167 |
| AX | 1.20 (2.02) | 1.26 (2.54) | 1.16 (1.74) |  | 0.412 |

Data are presented as n (%) or mean (SD). Current exposure refers to the last 12 months. Early exposure refers to the to 1^st^ year of life; BMI, body mass index; SPT, skin prick test; the *X^2^ test was used for comparing frequencies, the t test for comparing means.

**Table S1.** Subject characteristics of the random sub-sample *vs* the rest of the sample
